# Thermal pattern of Tatun volcanic system by satellite-observed temperatures and its correlation with earthquake magnitudes

**DOI:** 10.1038/s41598-023-47048-1

**Published:** 2023-11-10

**Authors:** Hai-Po Chan, Yu-Chang Chan, Cheng-Wei Sun

**Affiliations:** 1 Institute of Earth Sciences, Academia Sinica, Taipei, Taiwan; 2 Department of Geosciences, National Taiwan University, Taipei, Taiwan

**Keywords:** Environmental sciences, Natural hazards, Solid Earth sciences

## Abstract

The land surface temperature (LST) of volcanoes detected from satellite sensors reflects the thermal status of heat sources in the subsurface. Volcanic earthquakes occur as magma and volcanic fluids transport to the surface from depth. Thus, both LST and earthquake magnitude are key parameters to the study of active volcanoes. Here we investigate the volcanic status of Tatun Volcanic Group (TVG) based on LST and seismic observations. The Earth-observing satellites onboard thermal sensor derived land surface temperature, and the seismic records retrieved volcanic earthquake magnitude are used to delineate the past and current pattern of volcanic activity plus the future trend of the TVG. The spatiotemporal distribution of LST and volcanic earthquake magnitude in TVG are analyzed. The high-similarity trends of the 4-decade LST time series and 3-decade earthquake magnitude time series are inspected. The retrieved surface thermal pattern shows the non-steady-state nature of the subsurface thermal sources at this volcanic complex. The LST trend exhibits a rather positive correlation with the energy released from volcanic earthquakes and consequently, the presumption on the connection between LSTs and earthquakes is validated.

## Introduction

Volcanoes and earthquakes neighboring urban areas are apt to draw large attention from the public, especially in the modern metropolitan Taipei city of Taiwan. Since volcanoes and earthquakes generally pose a potential threat and high risk to human life and property, city governance must have the appropriate monitoring and mitigation management of those natural hazards. In early this year 2023 (Feb. 17, 2023), a magnitude M = 3.1 earthquake occurred just north of Taipei, which was likely caused by volcanic activity in the area of Tatun Volcanic Group (TVG). Due to the existence of a magma chamber under TVG, prior seismic research suggests that subsurface hydrothermal or volcanic activity is most likely the source of local seismicity in the TVG^1,2^. According to the report from the Central Weather Bureau (CWB) of Taiwan^3^, the earthquake is a shallow earthquake with a depth of 4.8 km only and considered to be triggered by the volcanic activity in the TVG. The possible explanation is that the magma reservoir under the volcano contains thermal fluid, which enters surrounding rock formations. That collapses off the stability of certain structures, causing rock movements that give rise to earthquakes. There have been 17 such earthquakes at that same location in the last 20 years.

**Figure 1:**
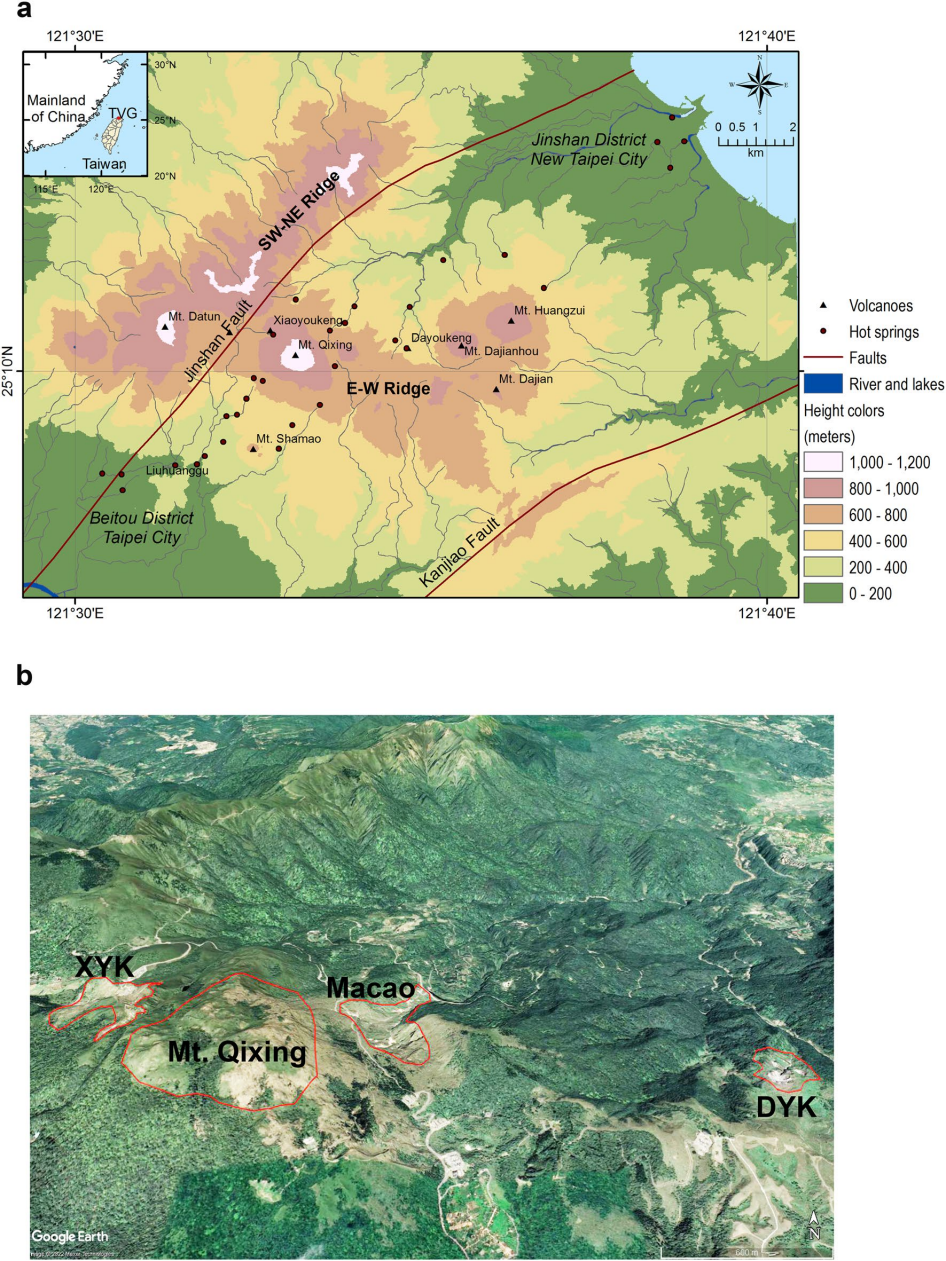
Geography and digital elevation model (DEM) map of Tatun Volcanic Group (TVG) of Taiwan. (**a**) TVG DEM color-ramp map based on the datasets provided by the Ministry of the Interior, Taiwan. (**b**) TVG 3D imagery from Google Earth. The four polygonal thermal anomaly regions of interest are illustrated, namely, Xiaoyoukeng (XYK), Mt. Qixing, Macao, and Dayoukeng (DYK).

The TVG lies 15 km north of Taipei's capital city and overlooks roughly 7 million people that live in the Taipei metropolitan area. It is composed of 23 Quaternary-age volcanoes covering approximately 113 km^2^ on the northern tip of the island of Taiwan. No historical eruptions have been recorded^4,5^. TVG was reserved as a part of Yangmingshan National Park in 1962. There is widespread evidence of apparent volcanic activity, including sulfur fumaroles, steam vents, and hot springs (e.g. Xiaoyoukeng, Dayoukeng, Liuhuanggu, etc.). As seen in Fig. 1, the TVG consists of E–W and SW–NE volcanic ridges between the Jinshan and Kanjiao faults. The average elevation of each ridge is between 800 and 1000 m above sea level, and each ridge is around 15 km long. Due to significant erosion, the SW–NE ridge has a sharper ridge and a steeper alpine. The E–W ridge, in comparison, has a much more rounded shape and still contains many of the primary volcanic structures, including cones, craters, lava flows, and pressure ridges^6^.

Previous seismological and geological work indicates the prominent extensional tectonics in TVG. A minor fault zone in TVG was identified based on the lineament map extracted from the digital terrain model, as well as aerial photos and field validation. The minor fault zone with a size around 3.5 km width by 16 km length is stretched in a NE–SW direction and generally aligns with the Jinshan fault's surface trace. The fault zone encompasses both tectonic fractures and hydrothermal activities in the TVG^7^. A model of volcano–hydrothermal system beneath the Mt. Qixing–Dayoukeng area was built based on the local geology and the seismicity features. The zone of highly fractured and fluid-saturated rock may lie underneath Mt. Qixing, extending to Dayoukeng in the upper 3 km of the crust. Estimates of the depth of the postulated magma chamber are deeper than 7 km^8^. Previous volcanic studies have delineates the heat transfer process in geothermal systems, in general, geothermal reservoirs (i.e. magma chambers) have numerous near-vertical faults and relatively impermeable intrusive interspersed in the aquifers. The thermal anomalies are detectable by surface manifestations, aerial or spaceborne infrared surveys, geochemical analyses, or exploratory drillings^9–11^. Two clustered seismic zones are identified in the TVG. One is located nearby Dayoukeng, and the other is beneath Mt. Qixing. Both seismic zones are likely triggered by the significantly volcanic gases and fluids raising from the magma reservoir in depth^2^. Based on findings from the research literature, the Mt. Qixing–Dayoukeng area in TVG can be the viable heat release conduit of the magma chamber and serves as the observation spot for the volcanic activities. Today, TVG is closely monitored by the Taiwan Volcano Observatory (TVO), which is responsible for assessing the volcanic hazards in the area and issuing warnings to the public in case of increased volcanic activity^12^.

Land surface temperatures (LST) reflect temperatures below the ground in a volcanic area. Volcanoes provide the subsurface heat source, channeled by the steaming vents, geysers, hot springs, lava flows, lava domes, etc., to form the thermal features on the land surface, and satellite sensors from space can detect land surface temperatures^13–18^. When volcanoes release heat, earthquakes near the heat source can be triggered by the moving magma and volcanic fluids, thus volcanic earthquakes are caused by liquid magma, hot fluids, and gases forcing their way through the crust to reach the surface^19–21^. Since both land surface temperatures and volcanic earthquakes are originated from thermal sources in a volcanic area, there is an evidential presumption that shallow volcanic earthquakes underground usually relate to the surface temperature on the ground. To validate the correlation between the surface temperature and subsurface earthquake at or near volcanoes is a demanding quest, because long-term records on temperature and earthquake at a specific volcano are required. The U.S. Landsat program since 1972 and Taiwan’s Central Weather Bureau Seismographic Network (CWBSN) since 1991 have provided valuable long-term records and datasets at TVG. Both institutions provide valuable long-term records and datasets at TVG. TVG thus provides researchers a unique opportunity to validate the presumption on the linkage between LSTs and volcanic earthquakes.

The objective of this work is to integrate satellite LST observations and ground seismic dataset to investigate the subsurface magmatic processes of active volcanoes, meanwhile, to validate the relationship between the LST and earthquakes in TVG. The thermal pattern of the TVG volcanic system by satellite-observed temperatures and its correlation with earthquakes are explored. We inspect its geothermal state based on 4-decade land surface temperature (LST). Two datasets of TVG are employed to define the past and current pattern of geothermal activity plus the future trend of the TVG. First, the U.S. NASA Earth-observing satellites onboard thermal sensor derived time series of land surface temperature from 1984, and second, the Central Weather Bureau Seismographic Network (CWBSN) of Taiwan seismograph records retrieved time series of shallow earthquake catalog from 1991. Long-term time series on LST and energy released from the volcanic earthquakes of TVG hotspots are analyzed and compared. Specifically, the 4-decade surface temperature and 3-decade subsurface earthquake-released energy are inspected and the possible connection between them are validated. To our knowledge, we are the first one to conduct this time series comparison between the LST and earthquake magnitude for volcanic study.

## Results

**Figure 2:**
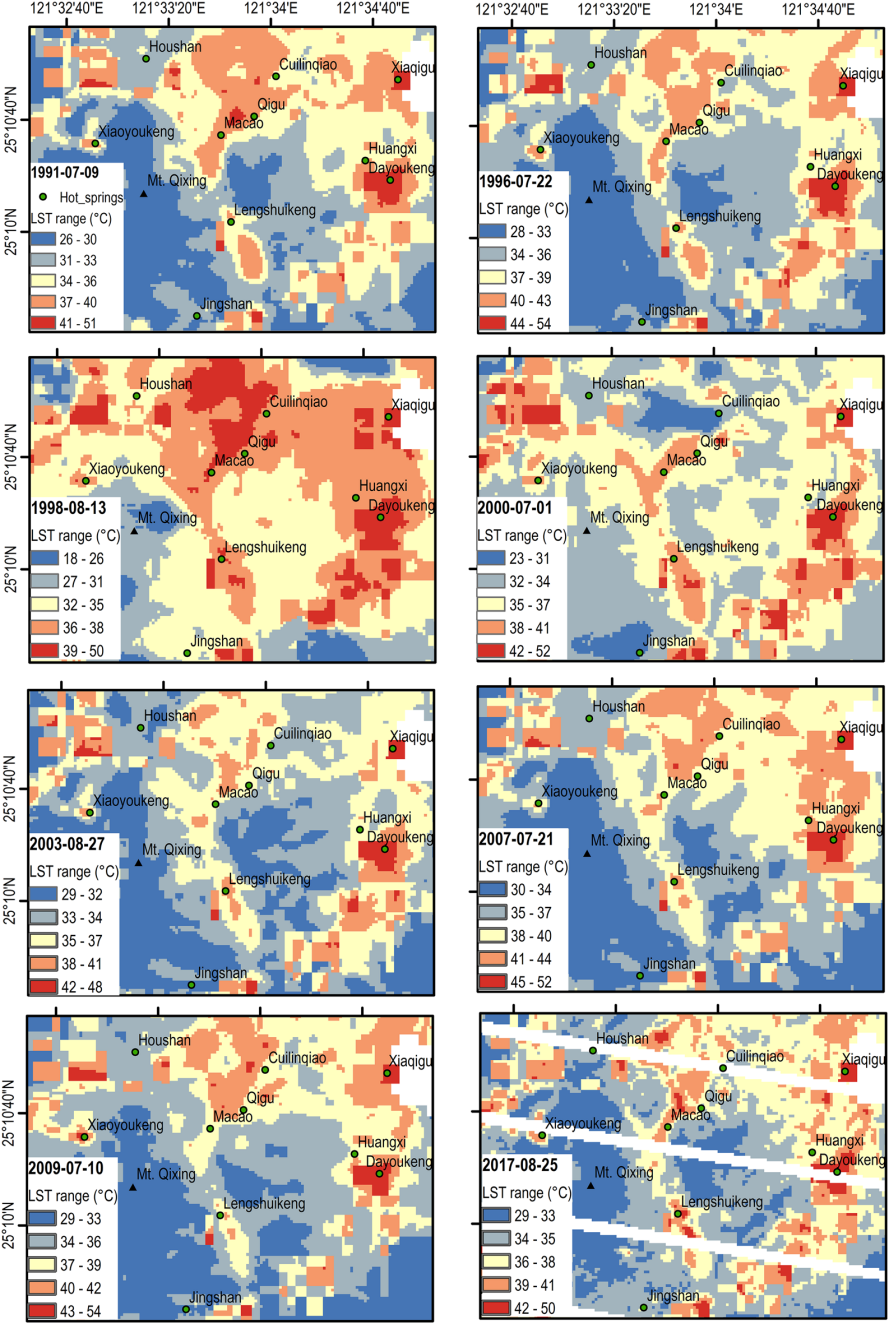
Spatial variations in land surface temperature retrieved from Landsat at TVG from 1991 to 2017. Note that the last imagery is contaminated by clouds plus sensor malfunction gaps.

Landsat Collection 2 Level-2 surface temperature science products have been used for the LST time series analysis. TVG has an area of around 400 km^2^, while the Landast LST imagery datasets have a 30 m spatial resolution, and therefore Landsat sensor is capable to collect detailed thermal activity features on TVG. However, it is not effective to analyze the LST of the whole group because the TVG is composed of 23 volcanoes. To elaborate on the thermal anomaly pattern on the land surface, we first need find a candidate to be the pilot volcano. Thus, a general view of temporal-spatial LST distribution in TVG is required to facilitate the selection. Figure 2 shows the exemplary multitemporal LSTs of the TVG from Landsat imagery selected from 1991 to 2017. Thermally anomalous regions are generally 3–15 °C above the surroundings. Anomalous thermal distributions are indicated by red colors.

**Figure 3:**
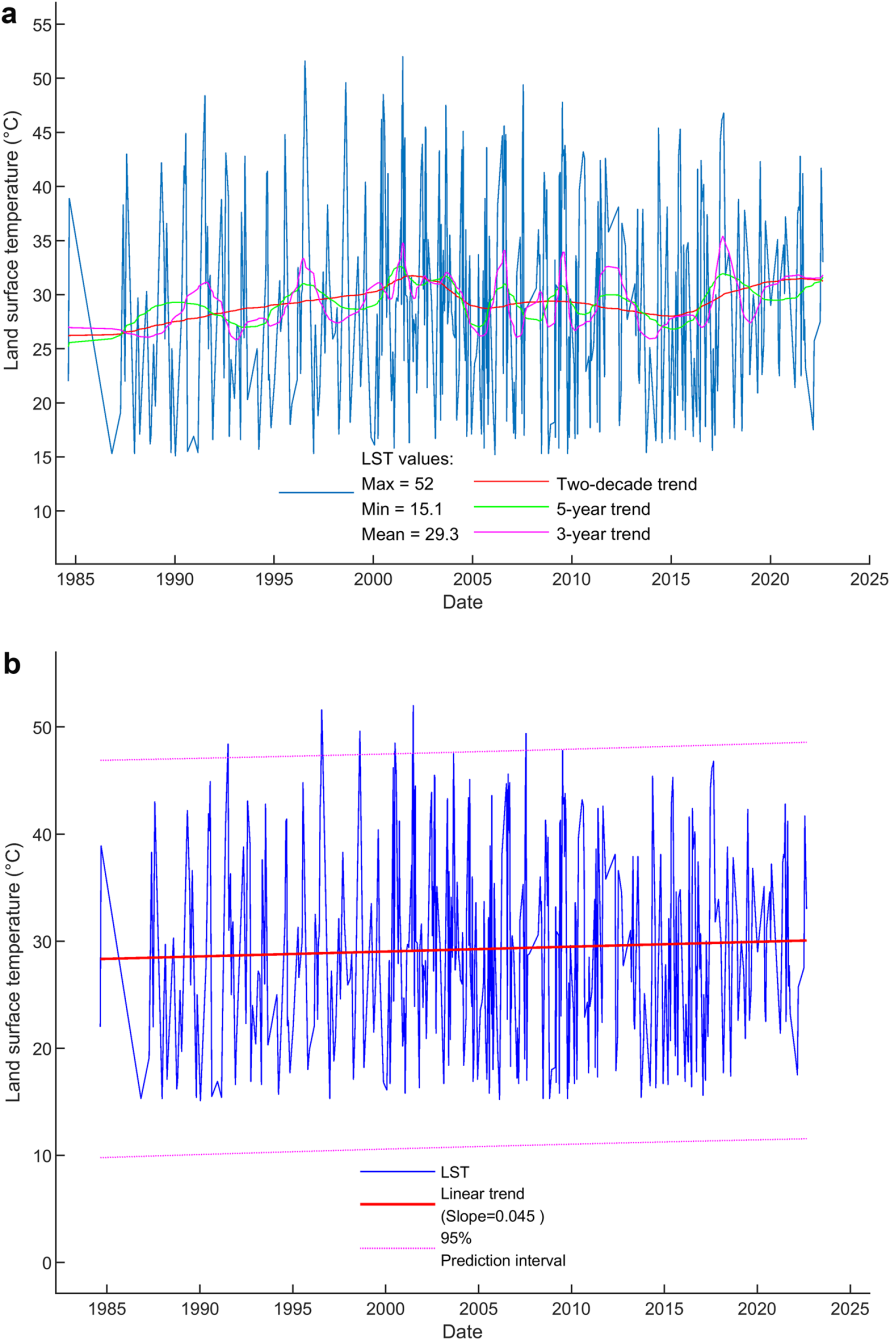
The LST time series and its trends at DYK from Landsat in the period August 1984 to September 2022. (**a**) The LST time series at DYK from Landsat (based on 415 data points) in the period August 1984 to September 2022. The simple oscillatory mode decomposition using EEMD was used to detect trend patterns over various time periods namely: the 3-year, 5-year and 2-decade trends. (**b**) The Landsat LST time series and its linear trend. The solid red line indicates the linear trend of Landsat LST time series (slope = 0.045 °C per year). Dotted lines indicate the 95% prediction interval.

Landsat images in Fig. 2 are contaminated with clouds and image gaps. However, the images show the clear consistency in thermal anomaly areas of TVG which are caused by the activities of the subsurface magma sources. Based on the pattern of LST distribution on TVG for Landsat imagery in Fig. 2, we have selected four geothermal sites to zoom in for further investigation, as shown in Fig. 1b, namely, Xiaoyoukeng (XYK), Mt. Qixing, Macao, and Dayoukeng (DYK). Among them, Dayoukeng (DYK) appears to have a higher and consistent thermal anomalous area, thus we have picked it up for the TVG representative hotspot for further analysis. The LST retrieval time series of the rest three hotspots will be included in the Supplementary Information section for reference. As the Landsat LST retrieval result, Fig. 3 shows the LST time series and its trends at DYK from Landsat in the period August 1984 to September 2022. An increasing trend of 2-decade KST temperature time series is revealed. The temperature uptrending rate is around 0.045 °C per year.

**Figure 4:**
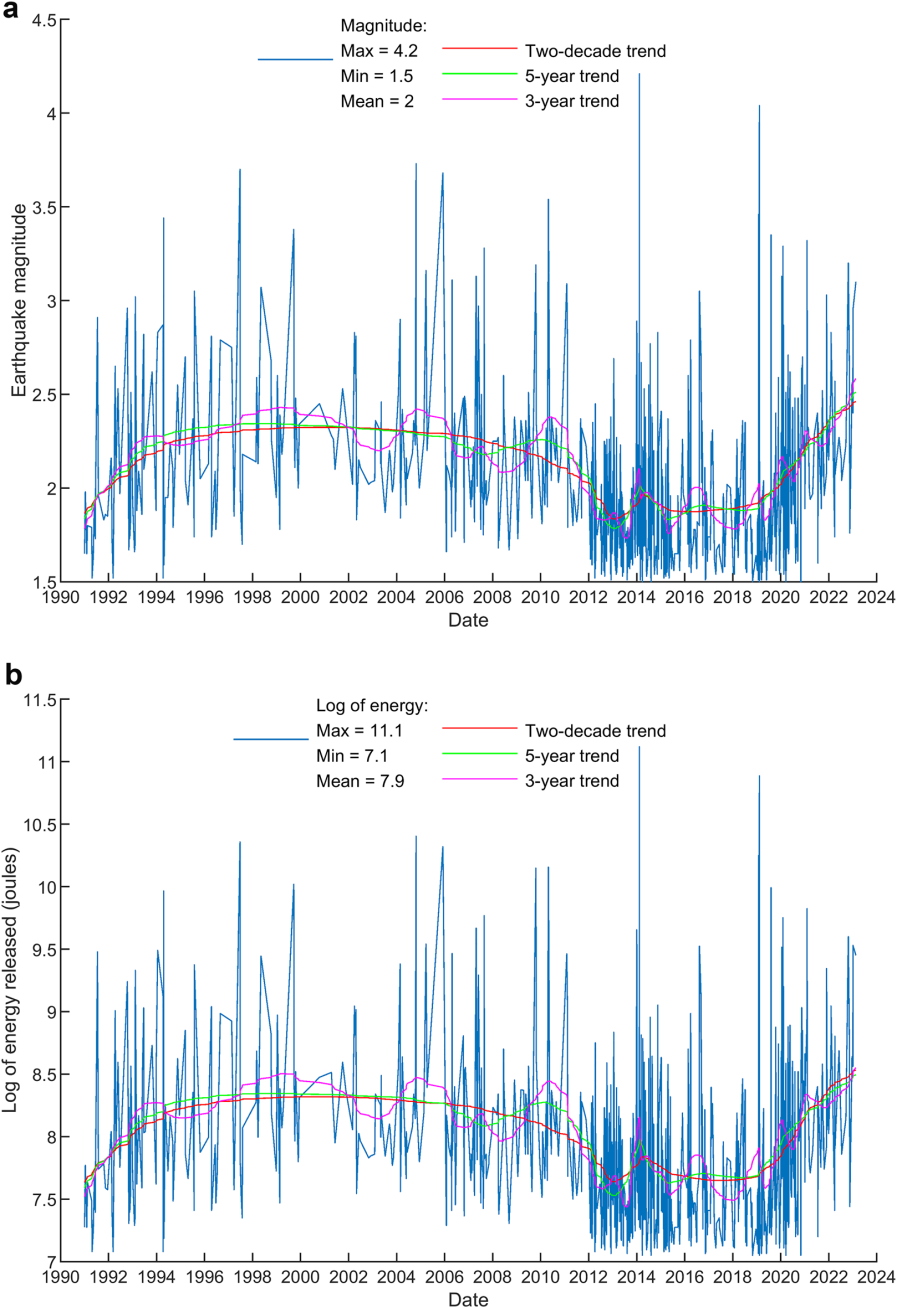
TVG shallow earthquake magnitude and earthquake energy released time series. (**a**) The earthquake magnitude time series (based on 692 data points). (**b**) The energy released form earthquakes is calculated from the magnitude.

Our next step is to retrieve the volcanic earthquake time series from Taiwan’s Central Weather Bureau Seismographic Network (CWBSN) seismograph records. The shallow earthquake catalog is retrieved from the Taiwan Seismological and Geophysical Data Management System^22^. The Taiwan Seismological and Geophysical Data Management System (GDMS-2020) is established through the collaboration between the Taiwan Central Weather Bureau (CWB) and the Institute of Earth Sciences, Academia Sinica (IESAS) in 2019. It is designed to serve the global research community with high-quality data, collected by the CWB seismic and geophysical networks. We have selected all available earthquake catalogs centered at DYK within a diameter of 20 km, depth is less than 30 km, and magnitude higher than 1.5. The criteria for selections is not arbitrary, but based on the results of previous research findings in TVG. Upon the retrieved earthquake magnitude, we have calculated the energy released from the earthquake magnitudes and sum it up on a daily basis. The earthquake energy release can be obtained by converting the magnitude to energy using the equation log E = 4.8 + 1.5 M, where M is the magnitude^23^. The resulting time series of earthquake magnitude and energy released from the earthquakes at TVG is illustrated in Fig. 4. Figure 4a shows the magnitude-time plot in TVG from CWB earthquake catalog. Figure 4b shows the log of calculated daily sum-up energy released from the earthquakes. They appear same pattern because the earthquake energy is decided by the big magnitude ones. Note that the numbers of earthquakes detected by the CWB increase suddenly after 2012. The reason of detectability inconsistency is that CWB updated all seismographs from 16-bits to 24-bits resolution in 2012^3^. The instrumental characteristics is one of the main factors to decide the detectability of a seismic network^24^. Acquisition of higher detectability seismic data generally results in better detection or resolution of minor earthquakes.

## Discussion

Recognition of the geothermal status quo of volcanism is important for quantitatively forecasting eruption potential at volcanoes. Previous studies^25,26^ use a cumulative volume curve to assess the reliability of the steady state model for an active volcano. However, only a small number of volcanoes in the globe have received adequate study to provide this information. In this study, we use the LST curve to assess geothermal status for the active volcanoes TVG. The criterion for a non-steady-state at a volcano is that the plot of the LST is non-linear with respect to time. In non-steady-state processes, mass and energy in the system are constantly changing with time. When a flow is non-steady, its characteristics—like pressure, velocity, or density—do depend on time^27,28^.

Both the LST and earthquake energy curves show the nonlinear pattern. LST’s nonlinear pattern indicates the non-steady-state activities of the subsurface thermal sources. Earthquake energy curve’s nonlinear pattern does not necessarily indicate the same. The reason is that LST and subsurface thermal sources are directly related via the sole temperature parameter. However, the relationship between earthquake magnitudes and subsurface thermal sources are much more complicated, and beyond the temperature parameter, but also depends on other parameters or mechanism yet awaiting to be discovered^29,30^.

**Figure 5:**
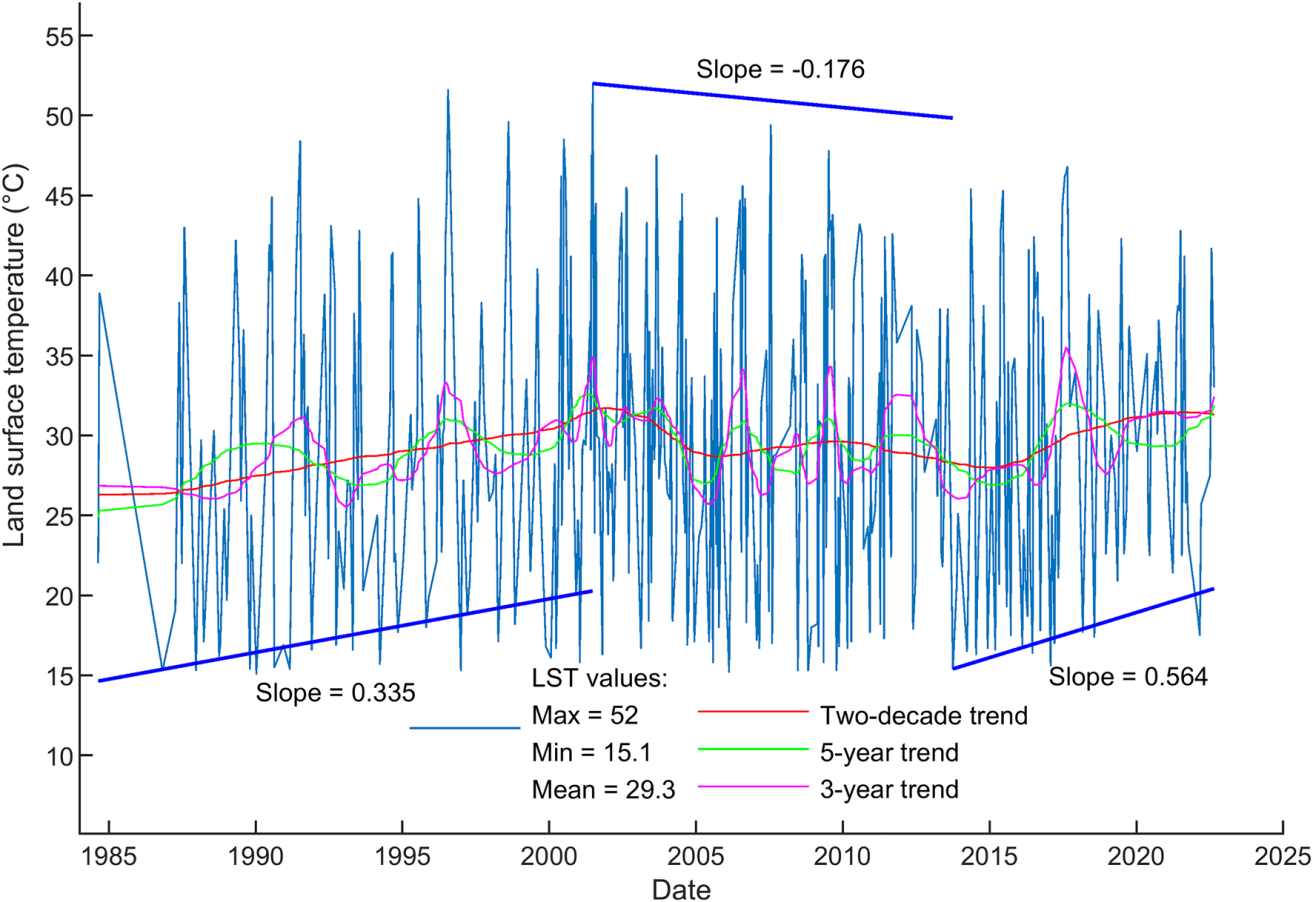
The step line chart of trend from Landsat LST time series. Three steps of the trend are recognized and marked with the slope of 0.335, − 0.176, and 0.564 °C per year, respectively.

**Figure 6:**
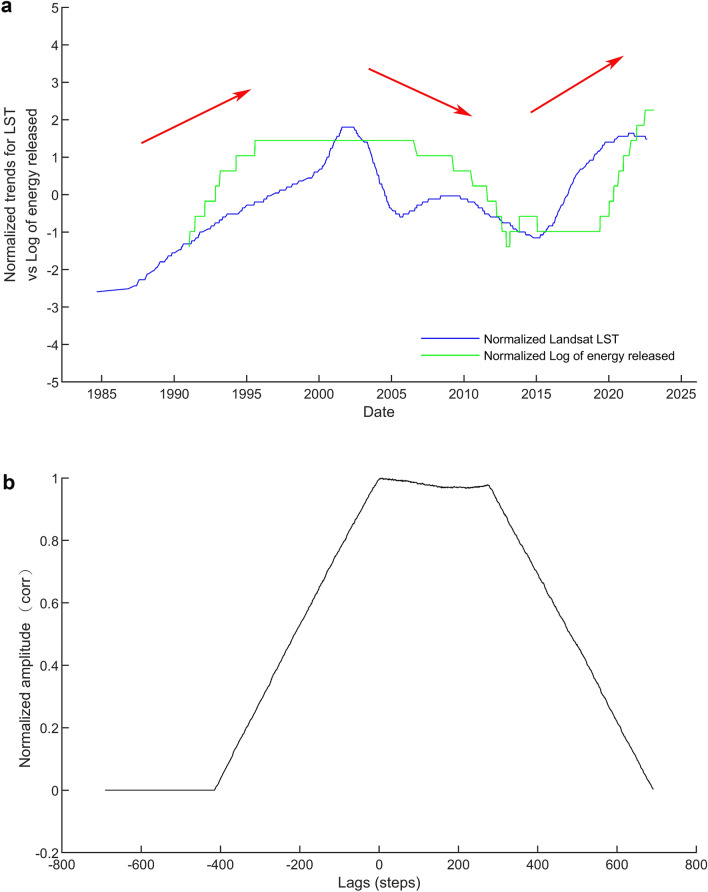
Comparison between the Landsat LST and earthquake energy released time series. (**a**) The normalization and comparison of trends from Landsat LST and earthquake energy released time series. Both time series are rescaled the data so they have normalized values with a mean of 0 and a standard deviation of 1. Red arrows indicate the up and down trending. (**b**) Cross correlation of the Landsat LST and earthquake energy released time series. The value of vertical axis (normalized amplitude; equivalent to corr) in the correlogram indicates the level of similarity. The time delay (lags) of the peak value is 8 days. It means delay LST by 8 days to match with earthquake occurrence.

To compare the trends from Landsat LST and earthquake energy released time series, we first make the step line chart of long-term trend from Landsat LST time series as shown in Fig. 5. Three steps of the trend are calculated and marked with the slope of 0.335, − 0.176, and 0.564 °C per year, respectively. For better comparison, we have extracted the long-term trends from both time series. Figure 6 shows the normalization and comparison of trends from Landsat LST and earthquake energy released time series. The similarity matching of curve pattern in Figs. 5 and 6 indicates the trends of LST and earthquake time series are consistent with each other. This comparison gives proof of the positive correlation between surface temperatures and subsurface earthquakes. We have mentioned in the beginning the presumption that volcanic earthquakes underground usually relate to the surface temperature on the ground. Results in this work prove this presumption holds true in the case of TVG.

Geological explanation on the positive correlation between the surface temperature and subsurface earthquake is readily obtainable in the rich literature of TVG. Previous studies show that TVG is a relatively young and energetic volcano with an active magma source less than 30 km depth. For instance, Magma chambers may be present beneath the TVG, according to a study on helium isotope ratios^31^. According to a study using TVG volcanic ashes, the most recent eruption with a VEI (volcanic explosivity index) of 4 may have occurred around 5500 years ago^32^. According to another study, lava, pyroclastic flows, and lahars are the three types of volcanic risks that could occur if Mt. Qixing continues to erupt as it has in the past^33^. According to seismic observations, a magma chamber is present beneath TVG, and a future eruption might occur^1,2^. Local seismicity in the TVG is believed to be triggered by the circulation of hydrothermal fluids from subsurface hydrothermal or volcano-related activities, according to analysis of shallow crustal earthquakes (depth less than 30 km)^8,34,35^.

Our results on the validation of the presumption on the connection between LSTs and earthquakes provide an alternative approach for volcano studies. It is convenient, intuitive and verifiable. Owning to the decades-long observations in remote sensing and seismology, the long-term records on temperatures and earthquakes at a specific volcano nowadays are available globally. Risk mitigation of attention-grabbing volcanoes generally requires constant monitoring and evaluation of the volcanic activity, and such studies demand long-term volcanic observation and quantitative analyses of long-period thermal behavior. Fortunately, remote sensing and seismic datasets offer a joint application for this need, and the integration gives an insight of hidden volcanic mechanisms to facilitate the mitigation of potential consequential hazards.

## Conclusions

The increasing trend of the 4-decade TVG temperature time series is revealed from Landsat satellite thermal images. The calculated linear temperature uptrending rates are 0.045 °C per year. The upward trends shown in the 4-decade LST time series signify the increasing intensity of thermal activities undergoing at the TVG. The LST curve pattern implies that the activities of the subsurface thermal sources are in a non-steady-state (transient) process. This study's satellite and ground seismic monitoring-based approach is applicable for exploring TVG's thermal status in the past as well as the future. Integrated application of long-term surface temperature and earthquake magnitude datasets is valuable for monitoring, hazard assessment, and mitigation of volcanic activity as well as for investigating the subsurface structure of volcanoes. This approach is favorable when access to the volcano is restricted or there is funding consideration for volcanic hazard management. In addition, it supports and fits with the existing volcano observatory systems, which will substantially improve the monitoring efficiency on the TVG.

### Methods and data set

Landsat imagery retrieved land surface temperature is extensively used in this study. A thermal remote sensing system generally uses a detector (sensor) to collect the thermal infrared energy that the earth's surface emits. Sensors record the energy as voltage, which an analog to digital converter turns into the scaled integers (called the Digital Number, or DN) for the energy^36,37^. Similarly, NASA satellite’s onboard thermal sensors acquire radiation energy from the land surface and convert the energy into the DN on the imagery (i.e., the grayscale images), and therefore the temperature can be converted from DN values to degrees Kelvin by the inverse of the Planck Function.

The thermal infrared bands on Landsat's sensors are in the 10.40–12.50 µm wavelength range. These wavelengths, which are utilized only to detect long-wavelength radiation emitted from the Earth and whose intensity mostly relies on surface temperature, are much beyond the range of human eyesight. The sensors' construction and operation are based on NASA-developed quantum mechanics principles^38^. Imagery from Landsat satellites and sensors, specifically Landsat 5 TM and Landsat 7 ETM + are used in this study. The Landsat satellites will pass overhead at 10 and 10.30 a.m. local time. 30 m and 16 days, respectively, serve as the spatial and temporal resolutions.

We have collected satellite thermal infrared images on the TVG for the period 1984–2022 from the NASA imagery archive in this study. Landsat Collection 2 Level-2 Science Products have been used for the LST time series analysis. Landsat Collection 2 includes scene-based global Level-2 surface reflectance and surface temperature science products. Collection 2 contains Landsat Level-1 data for all sensors since 1972, and Level-2 surface reflectance and surface temperature scene-based products from 1982 to the present. Each Landsat Collection is a set of methods for processing Landsat raw images. All of the data in a collection will have the same radiometric and geometric parameters, calibration methods, etc. As a result, users can make sure that a collection of Landsat images maintains a constant level of geometric and radiometric accuracy throughout time. While Level-1 comprises a scaled Digital Number (DN) (often 8 or 16 bit unsigned integers), Level-2 is atmospherically corrected data. Level-2 is the "ready to use data" with no need to implement any corrections. Collection 2 has improved data quality in comparison to collection 1. The absolute geolocation accuracy of Collection 2 is better than that of Collection 1, and it also includes updated digital elevation modeling sources, improved radiometric calibration, and improved quality assessment bands, as well as updated metadata and file formats. The Landsat surface temperature algorithm (Version 1.3.0), created in collaboration with the Rochester Institute of Technology and NASA Jet Propulsion Laboratory, is used to produce Landsat surface temperature products^39,40^.

The Ensemble Empirical Mode Decomposition (EEMD) is used to retrieve the trends from LST and earthquake time series. This work utilizes the EEMD to decompose the LST time series into several simple components, such as the simple oscillatory mode, in the time domain. The original LST time series are fully and orthogonally represented by the resultant LST decomposition components^41–45^. Thus, the trends of the LST time series are constructed by superimposing several simple EEMD components.

To elaborate in more detail on the EEMD, it is a robust empirical signal processing technique to handle nonstationary and nonlinear signals and extract useful information from them^43,46,47^. Most traditional signal processing techniques are based on linearity and steady-state assumptions. However, dealing with nonstationary and nonlinear signals at the same time is often needed in real-world circumstances. Specifically, for the alternative relations in the time, frequency, and energy domain, the EEMD is capable of handling nonstationary and nonlinear signals. The adaptive data decomposition technique termed EEMD is based on the characteristics of the data. The facts serve as the foundation for its analysis. Contrarily, the majority of common data decomposition techniques have an a priori foundation (for example, base wavelet in Wavelet transform and trigonometric functions in Fourier analysis), and hence are not adaptable^41^. The EEMD is used in practice to derive physical meaning from data, improve the understanding of physical events, and resolve engineering issues.

The goal of EEMD is to divide the Landsat LST time series into various components, each of which is connected to a different physical process. Particularly for the analysis of nonlinear and nonstationary data, the EEMD is appropriate. The essential component of EEMD is EMD, which allows any complex data set to be divided into a number of intrinsic mode functions (IMFs). Each IMF is an example of a narrow band frequency-amplitude modulation, which is often linked to a particular physical process. This decomposition technique is highly effective, flexible, and data-driven. Given that the decomposition is based on the local properties of the data, it is useful for nonlinear and nonstationary processes^41,42^.

The empirical and adaptive algorithm known as EMD allows a time series to be accurately expressed in the time–frequency–energy domain. When using EMD, the data x(t) are decomposed into IMFs, c_j_:

1a $$x\left( t \right) = \mathop \sum \limits_{j = 1}^{n} c_{j} \left( t \right) + { }r_{n} \left( t \right),$$

where

1b $$c_{j} \left( t \right) = { }a_{j} \left( t \right){\text{cos}}\left[ {\smallint \omega_{j} \left( t \right){\text{d}}t} \right],$$

and after IMFs have been extracted n times, r_n_ is the data residual of x(t). Through a sifting procedure that exclusively makes use of local extrema, the EMD is put into practice. The stages are as follows for any data set where x(t) = r_j−1_: In order to calculate the local mean, one must first take all local extrema (maxima and minima), connect them with a cubic spline as the upper (lower) envelope, and treat the resulting data as the new data. This process is repeated until the envelopes are symmetric about zero to a predetermined threshold. The final h is identified as c_j_. The filtering process is complete when the residue r_n_ turns into a monotonic function or when no more IMFs can be recovered using the aforementioned method. Following the application of EMD to eliminate all oscillatory components (riding waves), the secular trend of a time series is then determined. Since being established around 20 years ago, EMD has amassed thousands of citations from the numerous successful applications in various scientific and engineering fields^48^.

The frequent occurrence of mode mixing is one of the original EMD's fundamental flaws. To address this weakness, a noise-assisted Ensemble EMD (EEMD) approach is suggested. The EEMD is a significant advance over the original EMD, and it deals with the mean as the outcome after sifting an ensemble of signals with white noise added. The signal is the only component that survives the averaging procedure because the EMD is a time domain analysis approach; this signal is then used to represent the actual and more physically meaningful result^47^.

### Supplementary Information

Supplementary Information.

## Data Availability

The data that support the findings of this study are openly available at the following URL/DOI: https://lpdaac.usgs.gov; https://doi.org/10.7914/SN/T5.
