# Supplementary material for: Thermal pattern of Tatun volcanic system by satellite-observed temperatures and its correlation with earthquake magnitudes
Source: Sci Rep. 2023 Nov 10;13:19568. doi: 10.1038/s41598-023-47048-1 (PMC10638264; doi:10.1038/s41598-023-47048-1)
Supplement: Supplementary file 1 — Supplementary Information. [file 41598_2023_47048_MOESM1_ESM.pdf]

Thermal pattern of Tatun volcanic system by satellite-observed temperatures and its correlation with earthquake magnitudes

Hai-Po Chan [a*], Yu-Chang Chan [a] and Cheng-Wei Sun [b]

[a] Institute of Earth Sciences, Academia Sinica, Taipei, Taiwan; haipochan@g.ncu.edu.tw; yuchang@earth.sinica.edu.tw
[b] Department of Geosciences, National Taiwan University, Taipei, Taiwan; d08224004@ntu.edu.tw
* Correspondence: haipochan@g.ncu.edu.tw; Tel.: +886- 2-783-9910 ext. 1407

Supplementary Information

The LST retrieval time series of three other hotspots, namely, Xiaoyoukeng (XYK), Mt. Qixing, and Macao as shown in Figure A1, Figure A2, and Figure A3.

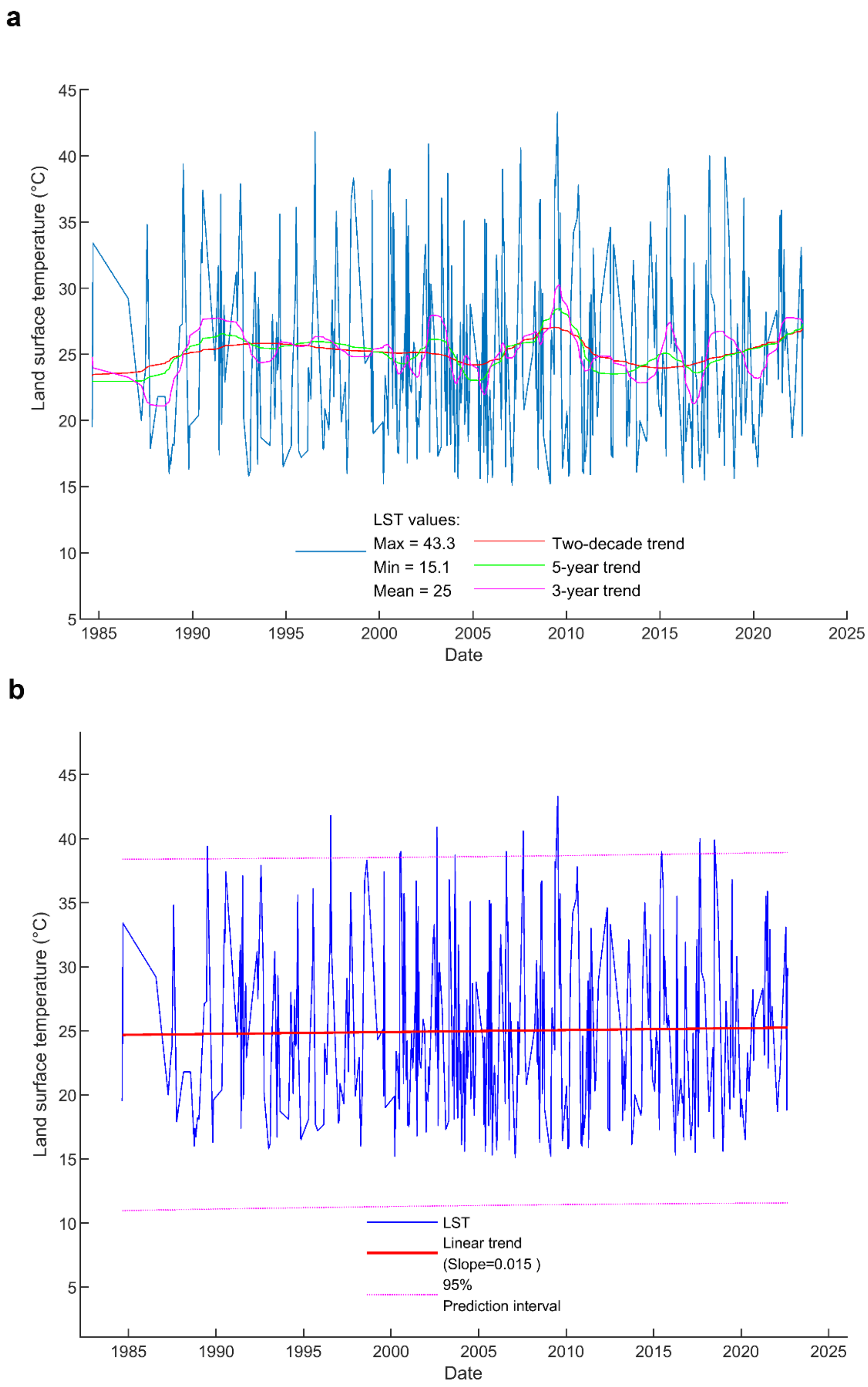

Figure A1. The LST time series and its trends at Xiaoyoukeng (XYK) from Landsat in the period August 1984 to September 2022. a) The LST time series at XYK from Landsat (based on 361 data points) in the period August 1984 to September 2022. The simple oscillatory mode decomposition using EEMD was used to detect trend patterns over various time periods namely: the 3-year, 5-year and two-decade trends. b) The LST time series and its linear trend. The solid red line indicates the linear trend of LST time series (slope = 0.015 °C per year). The 95% prediction interval is indicated by dotted lines.

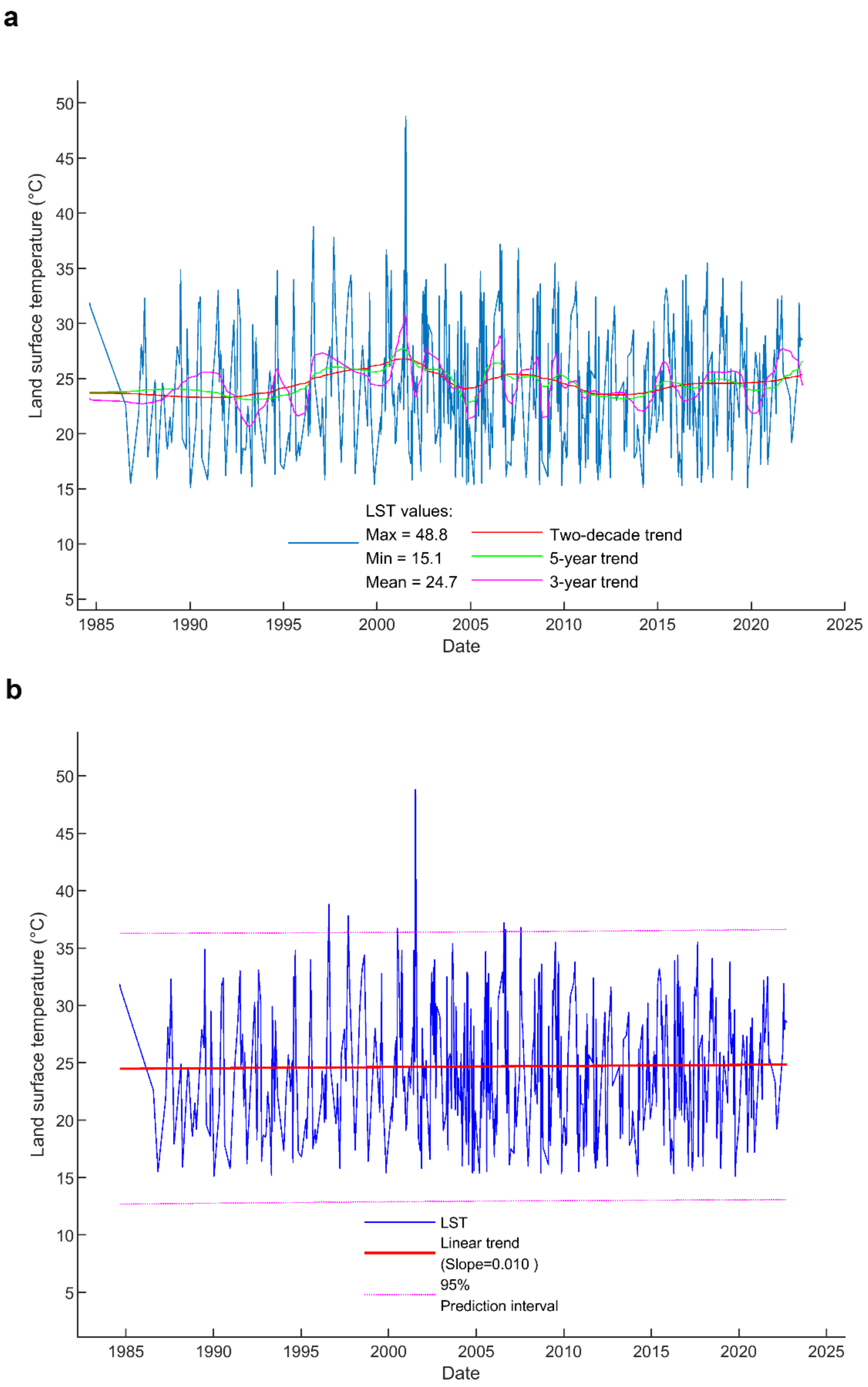

Figure A2. The LST time series and its trends at Mt. Qixing from Landsat in the period August 1984 to September 2022. a) The LST time series at Mt. Qixing from Landsat (based on 450 data points) in the period August 1984 to September 2022. The simple oscillatory mode decomposition using EEMD was used to detect trend patterns over various time periods namely: the 3-year, 5-year and two-decade trends. b) The LST time series and its linear trend. The solid red line indicates the linear trend of LST time series (slope = 0.010 °C per year). The 95% prediction interval is indicated by dotted lines.

a

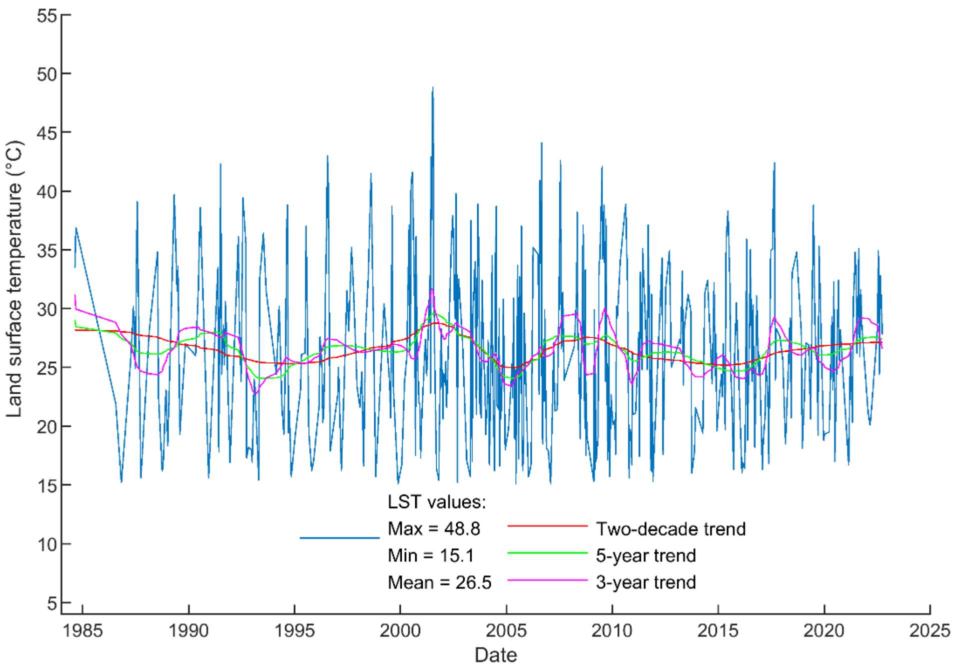

b

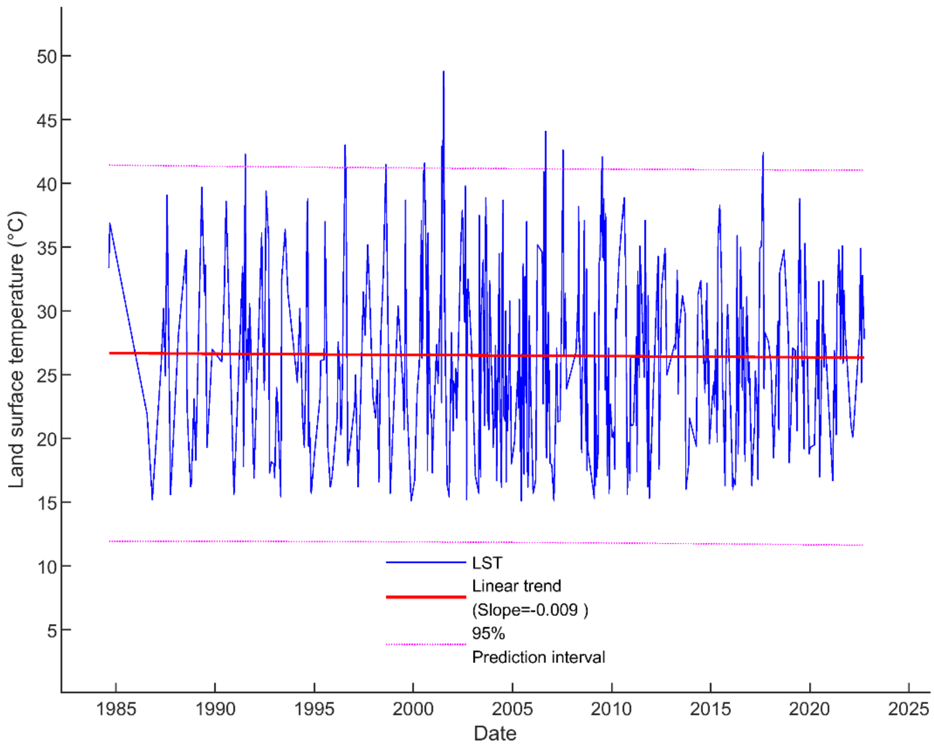

Figure A3. The LST time series and its trends at Macao from Landsat in the period August 1984 to September 2022. a) The LST time series at Macao from Landsat (based on 425 data points) in the period August 1984 to September 2022. The simple oscillatory mode decomposition using EEMD was used to detect trend patterns over various time periods namely: the 3-year, 5-year and two-decade trends. b) The LST time series and its linear trend. The solid red line indicates the linear trend of LST time series (slope = -0.009 °C per year). The 95% prediction interval is indicated by dotted lines.
